# Decreased Gaq expression in T cells correlates with enhanced cytokine production and disease activity in systemic lupus erythematosus

**DOI:** 10.18632/oncotarget.13903

**Published:** 2016-12-11

**Authors:** Yan He, Yan Huang, Lei Tu, Jiao Luo, Bing Yu, Hongyan Qian, Lihua Duan, Guixiu Shi

**Affiliations:** ^1^ Department of Rheumatology and Clinical Immunology, The First Affiliated Hospital of Xiamen University, Xiamen, Fujian, China; ^2^ Fujian University of Traditional Chinese Medicine, Fuzhou, Fujian, China; ^3^ Division of Gastroenterology, Union Hospital, Tongji Medical College, Huazhong University of Science and Technology, Wuhan, Hubei, China

**Keywords:** SLE, Gαq, T cell, apoptosis, Immunology and Microbiology Section, Immune response, Immunity

## Abstract

Aberrant T cell immune responses appear central to the development of systemic lupus erythematosus (SLE). We previously reported that Gαq, the alpha subunit of Gq, regulates T and B cell immune responses, promoting autoimmunity. To address whether Gαq contributes to the pathogenesis of SLE, Gαq mRNA expression was studied using real time-PCR in PBMCs and T cells from SLE patients as well as age- and sex-matched healthy controls. Our results showed that Gαq mRNA expression was decreased in PBMCs and T cells from SLE patients compared to healthy individuals. Correlation analyses showed that Gαq expression in T cells from SLE patients was associated with disease severity (as per SLE Disease Activity Index), the presence of lupus nephritis, and expression of Th1, Th2 and Th17 cytokines. In keeping with clinical results, T-helper cell subsets (Th1, Th2 and Th17) were over-represented in Gαq knockout mice. In addition, Gαq expression in SLE T cells was negatively correlated with the expression of Bcl-2, an anti-apoptotic gene, and positively correlated with the expression of Bax, a pro-apoptotic gene. These data suggest that reduced Gαq levels in T cells may promote enhanced and prolonged T cell activation, contributing to the clinical manifestations of SLE.

## INTRODUCTION

Systemic lupus erythematosus (SLE) is a multisystem autoimmune disease characterized by chronic immune activation, the presence of a plethora of autoantibodies, and diverse clinical phenotypes [1]. Although abnormal immune responses with excessive release of pro-inflammatory cytokines, as well as genetic factors, have been implicated in the pathogenesis SLE, the mechanistic details are still unclear [2, 3]. Recent compelling evidence has shown that abnormal Th1, Th2 and Th17 cell immune responses are crucial in the pathogenesis of SLE [4].

Members of the Gq family of membrane-associated heterotrimeric guanine nucleotide-binding proteins (G proteins) include Gq, G11, G14, and G15/16. They mediate the canonical activation of phospholipase Cβ isozymes, and like all heterotrimeric G proteins, are composed of three subunits, Gα, Gβ and Gγ [5, 6]. Gαq, the α-subunit of the Gq protein, is encoded by the GNAQ gene and is widely expressed in various cells of the immune system, including T cells [7, 8]. In recent years, by using knockout (KO) mice and chemical inhibitors, the functions of G proteins in the immune system have been extensively reported; accumulated data indicates that G protein signaling systems control important aspects of innate and adaptive immunity [9].

Several studies from our group and others have explored the relationship between Gαq signaling and autoimmune disease. Abrahamsen et al. reported, for instance, that stimulation of T cells with anti-CD3/anti-CD28 antibodies recruits Gαq subunits to lipid rafts, indicating that Gαq is involved in T cell receptor signaling [10]. Our own studies showed that migration from the skin to the draining lymph nodes after fluorescein isothiocyanate sensitization is impaired in Gαq-deficient (Gnaq-/-) neutrophils and dendritic cells [11], and that Gnaq-/- bone marrow chimeras with immune-specific Gαq deficiency spontaneously develop manifestations of systemic autoimmune disease with high titer antinuclear antibody, multi-organ involvement and swelling of the joints [12]. We also reported that the protein and mRNA levels of Gαq in peripheral blood lymphocytes from rheumatoid arthritis (RA) patients were significantly lower compared with healthy controls, and that decreased Gαq expression was closely correlated with disease activity [13]. Furthermore, Gnaq-/- T cells showed significant survival advantages both *in vitro* and *in vivo* [14]. These studies supported a pivotal role of the Gαq subunit in the pathogenesis of autoimmune diseases. However, whether Gαq contributes to the pathogenesis of SLE is not known. To address this question, Gαq expression was measured in peripheral blood mononuclear cells (PBMCs) and T cells from SLE patients, and its relationship with SLE Disease Activity Index (SLEDAI), clinical laboratory indicators, Th1, Th2 and Th17 cytokines, and apoptosis-regulatory proteins was determined. Our results showed a significantly decreased Gαq expression in both PBMCs and T lymphocytes from SLE patients, is in comparison with healthy individuals. In addition, significant correlations were observed between T cell Gαq expression and SLEDAI, Complement 3 (C3), and urine protein and creatinine (CRE) in SLE patients. As expected, Gαq expression was correlated with enhanced Th1/Th2/Th17 differentiation and cytokine secretion, and distinctly associated with the expression of the apoptosis-related genes Bcl-2 and Bax. Altogether, our data suggest that decreased Gαq expression might contribute to T cell dysfunction and development of SLE.

## RESULTS

### Decreased Gαq expression in PBMCs and T cells from patients with SLE

**Figure 1:**
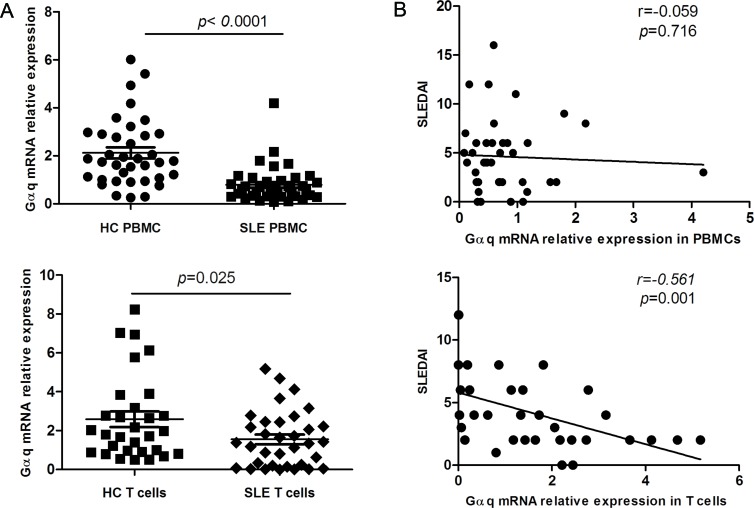
Decreased Gαq expression in PBMCs and T lymphocytes from SLE patients A. Expression of Gαq mRNA in PBMCs and CD3+ T cells from SLE patients and healthy controls (HC), detected by real time-PCR (SLE, n = 40, HC, n = 37). Each symbol represents an individual sample; horizontal lines denote median values. The Mann-Whitney U test was used to evaluate statistical differences between SLE and HC data. B. Correlation between T cell Gαq mRNA expression and disease activity index (SLEDAI) in SLE patients, assessed using the Spearman's rank correlation test.

A contribution of Gαq to the pathogenesis of RA was reported by us previously [13]. To assess if Gαq signaling is also associated with SLE, we first measured Gαq mRNA expression in PBMCs from SLE patients and healthy controls by real time-PCR. Although mRNA expression of Gαq was significantly lower in PBMC from SLE patients (Figure 1A, top) no correlation with SLEDAI was found (Figure 1B, top). Because T cells have been specifically implicated in the development of SLE, we next analyzed Gαq expression in T cells. As expected, the levels of Gαq mRNA in CD3+ T cells were lower in SLE patients than in healthy controls (Figure 1A, bottom), and correlated negatively with SLEDAI (Figure 1B, bottom).

### Correlation between T cell Gαq levels and parameters of disease activity

To assess the correlation between Gαq expression in T lymphocytes and organ involvement in SLE, SLE patients were grouped based on the presence or absence of renal damage (lupus nephritis), rash, arthritis, hematological involvement, serositis, oral ulcer, and alopecia (Table 1). Although for several parameters Gαq expression was lower in patients exhibiting clinical symptoms, a significant decrease in Gαq levels was found to be associated only with symptomatic lupus nephritis (p = 0.002; Table 1).

**Table 1:** Gαq mRNA expression in T cells from SLE patients with or without clinical manifestations

Clinical manifestation	YES n mean (Q1-Q3)	NO n mean (Q1-Q3)	p-value*
Renal damage	9 0.03 (0.01-0.30)	25 1.73 (0.84-2.59)	0.002
Arthritis	4 0.22 (0.06-0.71)	30 1.54 (1.17-0.25)	0.069
Rash	10 1.23 (0.06-0.71)	24 1.40 (0.29-2.52)	0.940
Low complement	24 1.36 (0.16-2.36)	10 1.50 (0.34-2.45)	0.587
Anemia	6 2.75 (0.15-4.80)	28 1.26 (0.17-2.20)	0.278
Thrombocytopenia	2 1.06 (0.07-2.06)	32 1.36 (0.22-2.44)	0.714
Leukopenia	3 2.23 (2.15-2.57)	31 1.33 (0.14-2.45)	0.192
Oral ulcer	2 0.88 (0.63-1.13)	32 1.40 (0.16-2.44)	0.558
Serositis	2 2.54 (2.18-2.90)	32 1.36 (0.16-2.44)	0.213
Alopecia	2 0.59 (0.05-1.13)	32 1.40 (0.22-2.43)	0.306

* Mann-Whitney U test was used to assess differences in Gαq expression in CD3+ T cells from SLE patients with or without clinical disease manifestations. p < 0.05 was considered statistically significant.

**Figure 2:**
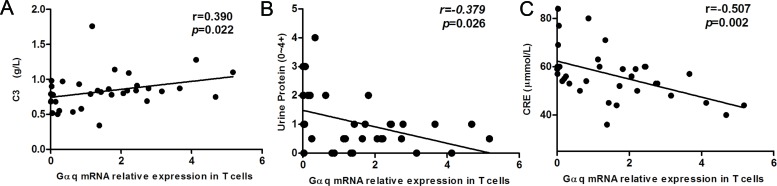
Correlation between Gαq mRNA levels in SLE T cells and laboratory values The relationship between T cell Gαq mRNA expression levels and laboratory values in SLE patients is shown. Spearman's correlation analysis was used to calculate significance.

Next, we analyzed the relationship between T cell Gαq expression and laboratory parameters in SLE patients. Gαq levels were positively correlated with C3 levels (r = 0.390, p = 0.022; Figure 2A). In line with the correlation found for lupus nephritis, negative correlations were detected between Gαq levels and 24h urine protein (r = -0.379, p = 0.026) and CRE (r = -0507, p = 0.002; Figure 2B, 2C). In contrast, no correlation was found between Gαq expression in T cells of SLE patients and dsDNA antibodies (p = 0.532), IgG (p = 0.970), C4 (p = 0.239) or ANA (p = 0.241; data not shown).

### Reduced Gαq expression is correlated with increased T cell cytokine expression and differentiation in SLE

**Figure 3:**
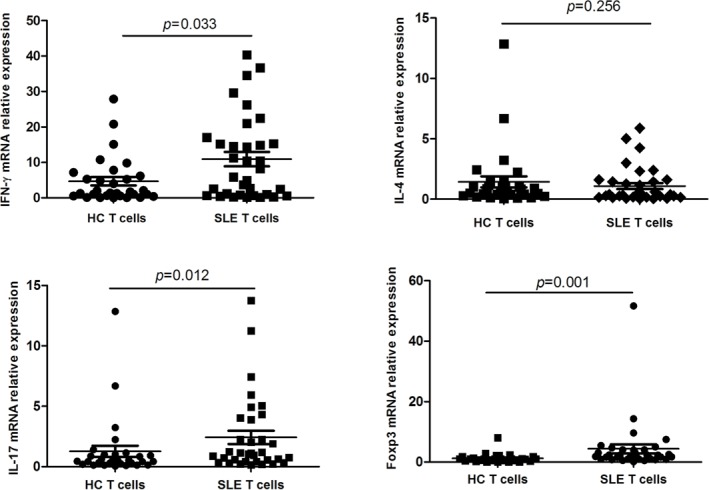
IFN-γ, IL-4, IL-17 and Foxp3 expression in T cells from SLE patients Relative mRNA expression of IFN-γ, IL-4, IL-17 and Foxp3 in CD3+ T cells from SLE patients and controls was detected by real time-PCR (SLE, n = 34; HC, n = 30). IFN-γ, IL-17 and Foxp3, but not IL-4, were increased in SLE T cells. Mann-Whitney U test was used to assess expression differences between SLE and HC.

**Figure 4:**
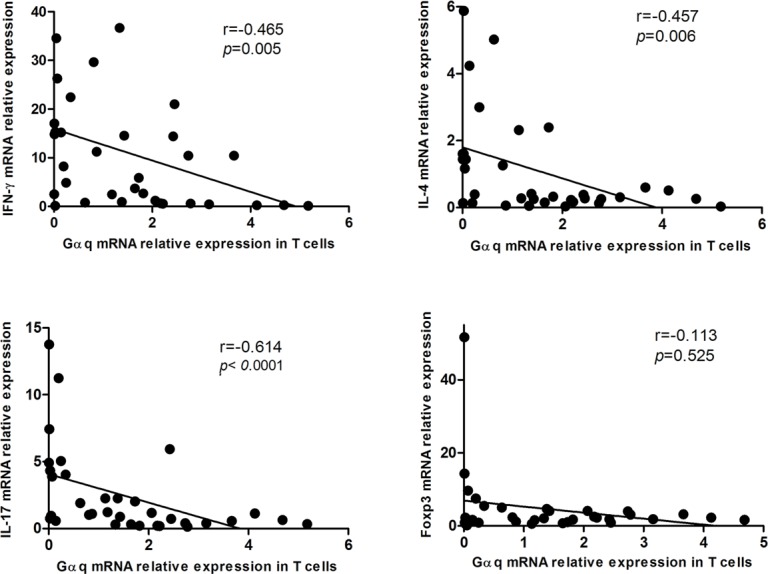
Correlation between Gαq and IFN-γ, IL-4, IL-17 and Foxp3 expression in T cells from SLE patients Spearman's correlation analysis showed that IFN-γ, IL-4, and IL-17, but not Foxp3, were inversely correlated with Gαq levels.

**Figure 5:**
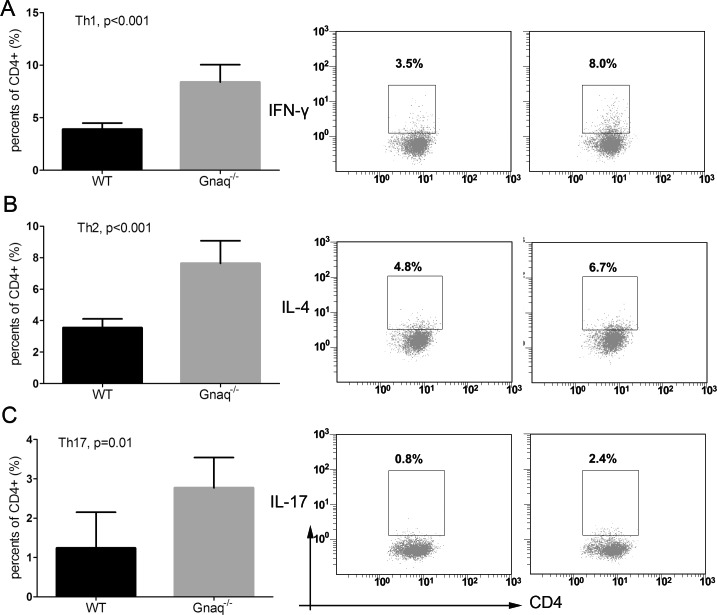
Gαq deletion promotes the differentiation of T-helper cells The expression of IFN-γ A., IL-4 B., and IL-17 C. was studied by flow cytometry in Gαq knockout (Gnaq-/-) and wild type (WT) mice-derived splenic CD4+ T cells stimulated with PMA, ionomycin, and BFA. Data from three independent experiments are presented as mean ± SD.

Because abnormal T cell activation is an important pathological feature of SLE, we further measured IFN-γ, IL-4, IL-17 and Foxp3 in T cells from SLE patients and from healthy controls. In keeping with previous studies, IFN-γ, IL-17 and Foxp3 expression was significantly higher in SLE patients than in controls (p = 0.033, p = 0.012, and p = 0.001, respectively), while no significant difference was observed in the expression of IL-4 (Figure 3). On the other hand, in SLE T cells a negative correlation between Gαq and both IFN-γ and IL-17 levels (Figure 4) was determined. In contrast with the corresponding findings in SLE patients and healthy controls, Gαq was negatively correlated with IL-4, but not with Foxp3 (Figure 4). To infer the impact of Gαq expression on T cell differentiation, splenic Th1, Th2 and Th17 subsets were quantified in Gαq knockout mice. As expected, the frequencies of CD4+ Th1, Th2 and Th17 cells were increased in knockout mice compared with wild type mice (Figure 5).

### Gαq levels and apoptosis-related gene expression in T cells from SLE

**Figure 6:**
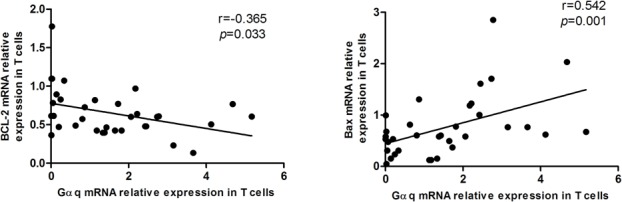
Correlation between Gαq and apoptosis-regulatory proteins in T cells from SLE patients Gαq mRNA expression is positively correlated with anti-apoptotic Bcl-2, and negatively correlated with pro-apoptotic Bax mRNA levels in SLE T cells. Spearman's correlation analysis was used to calculate significance.

An important feature of T cell dysfunction in autoimmune disorders is the prolonged cell survival that results from the abnormal onset and progression of the apoptotic program. Therefore, we analyzed apoptosis-related genes in T lymphocytes from SLE patients. As expected, a negative correlation between Gαq levels and the expression of the anti-apoptotic gene Bcl-2 was observed (r = -0.365, p = 0.033). In contrast, the expression of Gαq correlated positively with the expression of the pro-apoptotic gene Bax (r = 0.542, p = 0.001; Figure 6). These data suggest that Gαq might contribute to the pathogenesis of SLE by prolonging T cell survival.

## DISCUSSION

Gαq is abundantly expressed in the cells and tissues of the immune system [15]. Our previous work has demonstrated that Gnaq-/- chimeric mice possess several features of autoimmune disease, including production of autoreactive antibodies, deposition of IgG2a- and IgG2c-containing immune complexes in the kidney, thrombotic microangiopathy, a reduction in the number of red blood cells, synovitis, bone resorption, exostotic bone development, and osteolytic activity [12]. Furthermore, the involvement of Gαq in the pathogenesis of RA was confirmed by showing that Gαq prevented apoptosis in peripheral blood lymphocytes of RA patients by regulating the activity of Mcl-1 and caspase-3 [13]. Whether Gαq contributes to the pathogenesis of SLE, however, remains unknown. To address this question, the expression of Gαq was assessed in PBMCs and T lymphocytes from SLE patients. We found that Gαq expression was significantly decreased in both PBMCs and T cells from SLE patients, compared with healthy controls. However, only T cell Gαq mRNA expression was negatively correlated with SLEDAI, urine protein and CRE, and positively correlated with both lupus nephritis and C3 levels. These results suggest that decreased signaling through Gαq may be related to impaired kidney function in SLE.

The overproduction of Th2 cytokines typically promotes B-cell hyperactivity and humoral responses, while T cell hyperactivity and inflammation are frequently associated with an excess of Th1 cytokines [16]. The present data showed that the levels of IFN-γ (a Th1 cytokine), IL-17 (a Th17 cytokine) and Foxp3 (a marker of regulatory T cells) were higher in T cells from SLE patients, compared with controls, while low Gαq mRNA expression in T cells from SLE patients was associated with higher levels of IFN-γ, IL-4 (a Th2 cytokine), and IL-17. Moreover, intracellular staining showed that Th1, Th2 and Th17 helper cell subsets were over-represented in Gαq knockout mice, compared with wild-type controls. These data suggest that reduced Gαq signaling may contribute to augmented T cell activity in SLE.

Previous studies suggested that IL-17 contributes to the pathogenesis of kidney dysfunction in SLE patients [17–19]. We, on the other hand, have shown that Gαq inhibits the differentiation of Th17 cells by regulating the activity of extracellular signal-regulated kinase 1/2/(ERK1/2) to control the expression of STAT3 and RORα [20]. As significantly lower Gαq levels were measured in LSE patients with lupus nephritis, compared with those without renal damage, and low Gαq expression correlated with higher IL-17 levels in SLE T cells, our data may underscore a link between reduced Gαq expression in T cells leading to overproduction of Th17 cells, enhanced IL-17 production, and subsequent kidney damage.

Abnormal signal transduction in T-lymphocytes is considered a potential cause of lupus [21], with decreased MAPK activity and impaired ERK signaling among the important alterations found in T cells from SLE patients [22]. While several studies indicated that Gαq is involved in the activation of ERK [23], Gαq-/- primary T cells also show reduced proximal LAT phosphorylation, and in line with the present cytokine expression results, augmented immune responses, including increased secretion of IL-2, IL-5, IL-12 and TNF-α [24]. Another important hallmark of T cell dysfunction in SLE is apoptosis, a critical process for immune tolerance and autoimmunity [25]. The PI3K-Akt signaling pathway regulates many normal cellular processes, including proliferation, motility, and survival [26]. Gαq was reported to inhibit PI3K activation, which prevented Akt signaling and promoted apoptosis [27]. Accordingly, in a previous study we showed that upon T-cell receptor ligation, Akt activity was increased in Gnaq-/- T cells in comparison with wild-type T cells, and the survival advantage of Gnaq-/- T cells was significantly attenuated by Akt inhibition. We also proved that Gαq deficiency promotes T cell survival via upregulation of Bcl-xL and downregulation of Fas and FasL expression [14]. Here, we further show that Gαq expression was inversely correlated anti-apoptotic Bcl-2, and directly correlated with pro-apoptotic Bax mRNAs levels in T cells from SLE patients. These data further suggest that reduced expression of Gαq contributes to the pathogenesis and progression of SLE through inhibition or impairment of apoptosis in T cells.

In summary, we demonstrated that Gαq expression is significantly decreased in T cells of patients with SLE, and this is correlated with SLE disease activity, increased differentiation of Th1, Th2 and Th17 cells and altered levels of apoptosis-related proteins. Taken together, our results suggest that therapeutic restoration of Gαq levels may correct the over-excitable T cell phenotype in SLE.

## MATERIALS AND METHODS

### Patients’ characteristics, disease activity and clinical features

40 adult patients (35 women and 5 men, aged 32.6 ± 9.4 years) with a diagnosis of SLE based on the American College of Rheumatology criteria [28] were consecutively enrolled in the study after providing informed consent. All patients were referred from the Department of Rheumatology and Clinical Immunology at the First Affiliated Hospital of Xiamen University. Patients with cancer, hematopathy, severe infections, hepatitis, tuberculosis or allergies were excluded. The control group consisted of 37 healthy volunteers (34 women and 3 men, aged 33.5 ± 9.5 years) that were enrolled after giving informed consent. 34 patients and 30 controls donated additional blood for CD3+ T cell isolation. Table 1 depicts the demographic and clinical characteristics of all patients and controls. All clinical manifestations and laboratory findings were recorded on the day of blood withdrawal. Clinical manifestations of disease in SLE patients were determined on the basis of the SLE Disease Activity Index (SLEDAI), which assesses the presence of malar rash, alopecia, proteinuria, cutaneous vasculitis, oral ulcers, arthritis, and serositis [29]. This study was approved by the institutional research board (IRB) of the First Affiliated Hospital of Xiamen University.

### Animal experiments

All experimental procedures involving mice were approved by the Animal Care and Use Committee of Xiamen University. C57BL/6J (B6) and Gnaq-/- (backcrossed > 5 times to B6) mice were bred in the Xiamen University animal facilities and used between 6 and 8 weeks of age.

### Flow cytometry analysis

Spleen lymphocytes were harvested for intracellular cytokine staining. The lymphocytes were stimulated with 50 ng/ml PMA, 500 ng/ml ionomycin, and 5 μg/ml BFA (Alexis Biochemicals) followed by surface anti-CD4 staining. The cells were then fixed, permeabilized and stained with intracellular antibodies against IL-4, IL-17, and IFN-γ. All antibodies were purchased from Biolegend.

### Separation of peripheral blood mononuclear cells and purification of T cells

Peripheral blood samples from SLE patients and healthy volunteers were collected in anticoagulant tubes. Peripheral blood mononuclear cells (PBMCs) were isolated by standard Ficoll-Hypaque density-gradient centrifugation for 30 min, and washed twice with phosphate buffered saline before T cell purification by negative selection with the human T Cell Isolation Kit (Miltenyi Biotec, Bergisch Gladbach, Germany). The purity of T cells was > 95% as determined by flow cytometry.

### Reverse transcription-polymerase chain reaction (RT-PCR) and real-time PCR

Total RNA was extracted from PBMCs and CD3+ T cells by TRIzol™ Reagent (Invitrogen, Carlsbad, CA) and reverse-transcribed to cDNA with a Transcriptor First Strand cDNA Synthesis Kit, according to the manufacturer's protocol (Roche); RT- PCR was performed with a Bio-Rad System. mRNA expression levels of Gαq, IL-4, IFN-γ, IL-17, Foxp3, Bax, Bcl-2 and GAPDH were determined using a real-time quantitative PCR System (ABI 7500). The SYBR Green master (ROX) was purchased from Roche. Cycling conditions were as follows: 95°C for 10 min, followed by 40 cycles of 95°C for 15s and 60°C for 1min. Gene expression data was normalized to that of GAPDH and relative expression was calculated by the 2-ΔΔCt method. The following primer sequences were used in Table 2.

**Table 2:** primer sequences

Gene	Sequence	
GAPDH	F: 5′-AGCCACATCGCTCAGACAC-3′	R:5′-GCCCAATACGACCAAATCC-3′
Gαq	F:5′-TGGTGTATCAGAACATCTTCACG-3′	R:5′-CTCGAACTAATTGTGCATGAGC-3′
IFN-γ	F: 5′-AGCTCTG- CATCGTTTTGGGTT-3′	R:5′-GTTCCATTATCCGCTACATCTGAA-3′
Foxp3	F: 5′-CACTTACAGGCACTCCTCCAGG -3′	R:5′-CCACCGTTGAGAGCTGGTGCAT-3′
IL-4	F:5′-CACAAGCAGCTGATCCGATTC-3′	R:5′-TCTGGTTGGCTTCCTTCACAG-3
IL-17	F:5′-AACCGATCCACCTCACCTTG-3′	R:5′- TCTCTTGCTGGATGGGGACA -3′
Bcl-2	F:5′-ATGTGT GTGGAGAGCGTCAACC -3′	R:5′- GCATCCCAGCCTCCGTTATC -3′
Bax	F:5′-CCTTTTCTACTTTGCCAGCAAAC-3′	R:5′- GAGGCCGTCCCAACCAC-3′

### Statistical analysis

All data were analyzed using GraphPad Prism 5. Mann-Whitney U-test and Spearman's correlation analysis were used to calculate significance. P values < 0.05 were considered statistically significant.
